# Isometric Exercise for the Management of Lateral Elbow Tendinopathy

**DOI:** 10.3390/jcm12010094

**Published:** 2022-12-22

**Authors:** Dimitrios Stasinopoulos

**Affiliations:** Department of Physiotherapy, Faculty of Health and Caring Sciences, University of West Attica, Agiou Spyridonos 28, Egaleo, 12243 Athens, Greece; dstasinopoulos@uniwa.gr

The most common tendinopathy in the elbow area and one of the two most common tendinopathies in the upper limb is Lateral Elbow Tendinopathy (LET). Although it is easy to diagnose LET, the optimal treatment approach is yet to be determined. A conservative approach is usually followed by clinicians. An exercise program is usually the first-line treatment approach for LET, reducing pain and improving function in those patients [1].

In the last 10 years, isometric exercise has become popular in the management of tendinopathy. Early promising results in this regard were reports of immediate pain relief in patellar tendinopathy with isometric exercise [2,3]. The only study describing the immediate effects of isometric exercise in LET reported an increase in pain, not a decrease in pain, immediately after exercise [4]. However, trials are needed to examine the effectiveness of isometric exercise in short, intermediate, and long-term follow-ups. No previous systematic reviews have evaluated the effectiveness of isometric exercise in the management of LET alone. The aim of is article is not to determine the effectiveness of isometric exercise in the management of LET by conducting a systematic review. This editorial article introduces topics related to the use of isometric exercise in the management of LET.

The effectiveness of isometric exercise in the management of chronic LET has been examined in three studies [5,6,7]. Isometric exercise was applied as the sole treatment in two studies [5,6]. In the third study, isometric exercise was part of a supervised progressive loading exercise program [7]. According to the findings of the study by Vuvan et al. (2020), it is doubtful whether this form of exercise can be efficacious as a sole treatment [5]. The findings of Park et al. (2010) support the concept that isometric exercise undertaken early in the course of LET (within 4 weeks) as a sole treatment provides a clinically significant improvement [6]. The results of the trial by Stasinopoulos and Stasinopoulos (2016) support the finding that isometric exercise can be used as part of a progressive loading exercise program [7]. However, the time loading per session in the program, including isometric exercise, was 2.5 times more than the program that did not include isometric exercise.

However, the dosage of isometric contractions is based on clinical experience and varies among studies. The dose prescribed by Park et al. (2010) was 50 repetitions of 10 s holds, four times a day [6]. The participants were instructed to do exercises gently and without pain to increase the compliance rate and to reduce the potential of further injury to the affected tissues [6]. This protocol is considered too burdensome. Vuvan et al. (2020) recommended contractions based on a percentage of maximum voluntary contraction from 20% increasing up to 35% [5]. This is a well-described protocol but is too complicated. In the study by Stasinopoulos and Stasinopoulos (2016), the patients performed isometric contractions of wrist extensors for 45 s with the wrist in dorsal flexion, with 15 repetitions, daily [7]. In this study, the patients were told to continue with the exercise even if they experienced mild pain [7]. However, they were told to stop the exercise if the pain became disabling [7]. Mild and disabling pain was monitored by asking the patient to rate the pain on a visual analog scale (VAS) before and after treatment [7]. Mild pain was defined as below 4 on the VAS, whereas disabling pain was defined as above 8 on the VAS [7]. In the study by Stasinopoulos and Stasinopoulos (2016), the description of the isometric exercise dose was incomplete according to the parameters needed to develop an optimal isometric exercise protocol (see the next paragraph). Bateman et al. (2022) tried to develop an optimized physiotherapy treatment protocol for the treatment of people with LET [8]. They recommended that isometric exercises, as part of the exercise program, should be performed for up to 60 s with maximal resistance and repeated five times, once daily [8]. It was determined that the exercise should provoke pain to a level that the individual patient deemed acceptable to them [8].

To evaluate the effectiveness of isometric exercise in the management of LET, it is important to develop an isometric exercise protocol. The optimal isometric exercise protocol will be based on the following parameters:

1. The anatomical definition of the exercise;
2. Load (maximal voluntary contraction of wrist extensors);
3. Repetitions;
4. Contraction mode and duration per repetition;
5. Rest between repetitions;
6. Sets per session;
7. Rest between sets;
8. Sessions per week;
9. Rest between exercise sessions;
10. Duration of exercise program.

Patients with LET also have reduced proprioception, in addition to pain and reduced function (see the first paragraph) [9]. Therapists ignore reduced proprioception in the management of LET [10]. Reduced proprioception delays the healing process. If physiotherapists use modalities to improve proprioception, the results will be effective sooner. Isometric exercise increases proprioception [10].

The mechanism by which isometric exercise provides pain relief in tendinopathy is not yet fully understood and should be clarified in the future. For the time being, isometric exercise is associated with a reduction in motor cortex inhibition [2].

It is proposed that isometric exercise be used at the beginning of treatment to reduce and manage tendon pain, increasing the strength at the angle of contraction without producing inflammatory signs [11]. Based on the literature, isometric exercise does not appear to be superior to isotonic exercise in the rehabilitation of chronic tendinopathy [12]. Future studies are needed to confirm this finding in the management of LET. Isometric exercise can be used as part of a supervised progressive loading exercise program or in a clinical placement exercise program, as it may be beneficial for selected individuals [12]. The latest recommended findings will be examined in future studies regarding patients with LET. However, all of the findings regarding isometric exercise in the management of LET will be confirmed when the optimal isometric exercise protocol is developed.
